# Supplementary material for: PET/CT targeted tissue sampling reveals virus specific dIgA can alter the distribution and localization of HIV after rectal exposure
Source: PLoS Pathog. 2021 Jun 1;17(6):e1009632. doi: 10.1371/journal.ppat.1009632 (PMC8195437; doi:10.1371/journal.ppat.1009632)
Supplement: Attachment — Submitted filename: Response to reviewers comments.pdf [file ppat.1009632.s002.pdf]

Northwestern Medicine
Feinberg School of Medicine

Thomas J. Hope, PhD
Professor
Principal Investigator
Cell and Developmental Biology
Northwestern University
Feinberg School of Medicine

312.503.2337 office
312.908.2528 fax
312.503.1360 direct
thope@northwestern.edu

Simpson Querrey, 6th Floor
303 East Superior Street
Chicago, Illinois 60611

Northwestern

Dear Editor,

We are pleased to resubmit this article entitled "PET/CT targeted tissue sampling reveals virus specific dIgA can alter the distribution and localization of HIV after rectal exposure" (PPATHOGENS-D-21-00483 - [EMID:8a75f474f28f074e). We would like to thank the reviewers for their thoughtful comments to improve the manuscript.

We believe that the revised manuscript and figures address the concerns and comments of the reviewers. We have changed the language of and added sections of the introduction, discussion, and methods for clarity. Specifically, there is a more complete description of the viral stocks including the rational for utilizing HIV-BaL virions, the super-physiological challenge dose, and the implications of these experimental conditions on the observed outcomes. Figures 3 and 5 have been revised by increase in brightness and the addition of increased magnification inserts increasing accessibility through helping the reader to easily see the individual particles within the tissue sections. We hope that the manuscript is significantly improved and is acceptable for publication in Plos Pathogens.

Responses to specific reviewer comments are presented in italics below.

Part I - Summary
Please use this section to discuss strengths/weaknesses of study, novelty/significance, general execution and scholarship.

Reviewer #1: Roslyn Taylor and colleagues used PET and fluorescence imaging to visualize the transmission and dissemination of HIV through the rectal route in non-human primates in the absence and presence of dimeric IgAs. The most surprising finding is how deep viruses penetrate within just two hours. In the presence of IgA viruses, virus entrapment in tissues increases, particles reach deeper and even arrive at the draining mesenteric lymph node. The authors observe interesting differences between IgA1 and IgA2. The hypothesis is that tissue retention and penetration are mediated by immune cells that capture viruses. These features are likely mediated by distinct Fc receptor expressing cells in various tissues.

Technologically the work is very elegant as it combines non-invasive PET with fluorescence imaging with both labeled virus and labeled IgAs. That the virus labeling method leads to reduced infectivity is likely not so important as these initial events are likely dominated by particle transport. This report clearly increases the appetite for more, but given the complexity, cost of the in vivo work, its pioneering nature, I do support publication in PLoS Pathogens. It is also highly relevant for the development of an effective vaccine that provides effective mucosal immunity.

We appreciate the supportive words relating to our "very elegant" technology and its current and future potential impact. We believe a better understanding of the impact of anatomy and physiology on virus-host interactions, utilizing methods such as PET/CT guided necropsies, are needed to solve the problems of vaccine development and other prevention approaches.

Reviewer #2: This is a very interesting - though preliminary - study of early viral dissemination in the GI tract

in the presence of dimeric IgA (dIgA), using combined PET-CT imaging and fluorescence microscopy. The study, performed in rhesus macaques, utilizes Cy5-conjugated dIgA with specificity for HIV Env, and 64Cu-labeled HIV expressing a photoactivatable form of GFP. The virus is therefore both fluorescent and radioactive, and the antibody is fluorescent but distinguishable from the virus. It is worth bearing in mind that HIV replication is severely restricted in rhesus macaques due to host factors, so the system described here has very real limitations and cannot be used to study the impact of virus-antibody interactions on the disease process. As a tool for assessing early viral dissemination without replication, though, the system seems to work well enough to make preliminary observations. The study addresses a limitation of previous virus localization studies that relied on fluorescence microscopy: the detection of fluorescence in situ is limited by tissue opacity, whereas detection of radioactive signal is minimally impacted by tissue thickness.

The findings as described in the text are intriguing and worthy of further study, particularly in light of the previous observation (referenced in the text) that HGN194 dIgA1 protected 5/6 animals from a high dose SHIV challenge. The role of sIgA in sequestering mucosally-acquired virus and perhaps facilitating its transport (as implied here) to MLN, and eventual antigen presentation, is quite intriguing and warrants additional follow-up.

Unfortunately I found several of the Figures unconvincing as currently presented; red virions are quite difficult to spot in these images; perhaps software is more effective than the naked eye.

The system utilized here is specifically designed to define and characterize the interaction of viral particles in the inoculum of exposure with the natural mucosal tissues within the native anatomy and physiology of a living non-human primate. It is designed for that purpose and limited to that purpose. This is a technical achievement, not a preliminary observation. The disease process requires viral replication. The marker PA-GFP-Vpr, critical to visualize individual virions within this highly autofluorescent mucosal environment, is lost with replication because it was provided in trans during virion production. Other methods must be used to study the impact of virus-antibody interactions on the disease process and that work is in progress.

Reviewer #3: Manuscript by Taylor et al evaluates the ability of PET/CT scanning to evaluate the ability of dIgA to influence the distribution of HIV after rectal exposure. The manuscript takes a highly innovative approach to ask an important question regarding the interplay between the HIV virus and a mucosal antibody at an important mucosal site.

Part II – Major Issues: Key Experiments Required for Acceptance
Please use this section to detail the key new experiments or modifications of existing experiments that should be absolutely required to validate study conclusions.

Generally, there should be no more than 3 such required experiments or major modifications for a "Major Revision" recommendation. If more than 3 experiments are necessary to validate the study conclusions, then you are encouraged to recommend "Reject".

Reviewer #1: (No Response)

Reviewer #2: - HIV replication is restricted in rhesus macaques due to host factors, so the system described here has very real limitations and cannot be used to study the impact of virus-antibody interactions on the disease process, or indeed anything much beyond initial dissemination of inoculum. Some discussion of these limitations (which are not addressed at all in the current text) would be helpful and would help put the study in perspective. In the current draft there is practically no discussion of the virus stock although it is said to be HIV BaL and not SHIV-BaL. (And the Discussion section talks about this study and earlier work, e.g., Ref. 17, without addressing this important difference).

Thank you for your comment. To clarify the details and implications of the use of PA-HIV-BaL in the studies presented here, we have added the following two sections and additional text to clarify how the viral particles are generated.

"The ability to efficiently load HIV-BaL with PA-GFP-VPR and the ability of the HGN194 binding specificity to neutralize HIV-BaL make this well-defined virus system an acceptable substitute for the SHIV-1157-ipEL-p utilized in the challenge studies (20). The inability of the HIV-BaL to infect macaque cells is not relevant in the short, two-hour duration of virus distribution analyzed." (page 8, lines 160-164)

"For these studies we utilized a PA-GFP labeled replication competent HIV-BaL, which has important implications. Utilizing PA-GFP-Vpr allows robust labeling of individual particles to facilitate the detection of individual particles that are efficiently bound by the various HGN194 isotypes in our study. We are not currently able to efficiently label SIV Gag based particles with tagged SIV VPR. The PA-GFP-BaL cannot infect potential target cells in the rhesus macaque model because of host restriction factors such as TRIM5a (Ref). The use of PA-GFP-BaL also allows us to compare outcomes to other studies defining virion distribution is NHP and explant culture models."(page 18, lines 364-370)

- The virus in these immune complexes is most likely targeted for degradation and/or antigen presentation, particularly if the so-called "mystery" cells are APCs. It would be interesting to use this model to track what happens next (degradation, Ag presentation), for example within the MLN. Do sIgA-mediated immune complexes, and their transport to MLN, enhance presentation of viral antigen, perhaps leading to enhanced mucosal immune responses?

Thank you for your comment. This is a great question that is of interest to us. We agree with the reviewer that these observations with the mystery cells and immune complexes open up possibilities of antigen physiology and trafficking which we hope to address in the future. While these experiments would be interesting, they require the development of new methodologies and are outside of the scope of this paper.

- How the challenge doses compare to the amount of virus that would be "received" during a sexual encounter is not clear.

The dose that was used in these experiments is overwhelming, which allows us to map where individual virions distribute after rectal challenge. The goal of our study is to examine where the virus goes after rectal challenge requiring a large amount of virus. To help the reader appreciate this issue we have added the following text.

"It is also notable that the amount of virus in this model is super-physiological compared to what is speculated for human transmission (discussed in 25). Large amounts of viral particles are needed for efficient tracking. However, the antibody amounts are also super-physiological as are the single large dose challenge approach utilized to identify the synergistic blocking activity of HGN194 dIgA2 and HGN194 IgG1 to block SHIV-1157-ipEL-p." (page 18, lines 370-375).

- As the authors point out, it will also be important in future studies to include IgG1 to determine its mechanistic impact.

Experiments that include IgG1 with IgA are planned for studies in the near future, but are beyond the scope of the current manuscript.

- I have looked long and hard at Figure 1D, but I only see one very faint red signal at the end of one of the white arrows; I don't see any others. Can a better image or exposure be found? Also, the images in Figures 3

and 5 are not particularly convincing for red fluorescence, with a few exceptions.

Thank you for your comment. We have brightened the entire image (~20%) while maintaining linear scaling in the revised in Figure 1D to help the reader with the visualization of the Cy5 signal (red) revealing IgA localization. We have also brightened the entire image (~20%) while maintaining linear scaling and created increased magnification insets to help the reader visualize the red viral particles of each microscopic image in the revised versions of Figures 3 and 5.

Reviewer #3: While the study was well designed and the technology is impressive, it is not clear that the data presented fully backs up the conclusions that are reached, this is in part due to the difficulties associated with the microscopic images. The issues:

1. Microscopy images are from frozen tissue, this is necessary to see the fluorescent signals from virions and antibodies (Figs 1,2,3 and 5). However, this makes it difficult to see the virions in the sections. The small white arrows are likely pointing to virions (I presumed this as I did not see where it was stated in text or figure legends) yet seeing them by eye still proved difficult. As the graphs in figures 4 and 5 are dependent on these data this difficulty reduces confidence in the graphs and therefore in the overall conclusions from the manuscript.

Thank you for your comment. Reviewer #1 also had difficulties visualizing the viral particles. To improve the presentation of the micrographs for the reader we have brightened all the images equivalently (~20%) while maintaining linear scaling which will assist the reader with the visualization of details. To facilitate visualization of individual particles, we generated increased magnification insets to help the reader visualize the red viral particles of each microscopic image in the revised versions of Figures 3 and 5. We believe that this representation of the virions that were found in the rectum, colon, and mesenteric lymph nodes will help the reader appreciate the graphic presentation of the quantified data.

2. The background green fluorescence seems high. Can the background fluorescence be turned down so that we can better see the IgA or the virions?

Thank you for your question. The green background fluorescence is a function of the tissue being imaged and represents a major obstacle to fluorescence imaging of tissue sections. It was because of this background issue that we developed the PA-HIV-BaL system in the first place. This background is leveraged as a part of our approach to utilize PA-GFP to identify individual virions in our images. We use the background to decipher differences between our pre- and post-activation of the photoactivatable GFP BaL in determining whether or not something is a virion. This is described in detail in reference 23 and had been utilized to follow individual viral particles in multiple tissues as previously published (female reproductive tract, penile tissue, oral exposure). Importantly, in these studies the PA-HIV-BaL functions to validate the PET signal.

3. A number of interesting and unexpected attributes were associated with the IgA administration. Is there any way to quantify the frequency of virions that are near IgA staining and to determine if IgA binding is influencing the depth of penetration or proportion penetrating.

The binding of dIgA1/2 and viral particles is a dynamic process. Therefore, we felt the best way to determine the impact of either HGN194 dIgA1 or HGN194 dIgA2 was to compare virion localization as we have done in this manuscript. The majority of the virions found in animals that received pre-labeled IgA-Cy5 were near IgA signal, but control virions had no IgA. Therefore, we utilized the quantification of the distribution and depth of the virions to reveal differences in the impact of HGN194 dIgA1 and HGN194 dIgA2 in regard to depth of penetration and proportion of penetrating virions (as shown in figures 4 and 5). In the transverse colon, we found that virions in animals that received dIgA2 penetrated deeper into the epithelium than dIgA1

(Fig 4C). In the rectum, dIgA1 resulted in a higher proportion of penetrating virions compared to PBS controls (Fig 4B). In the descending colon, both dIgA1 and dIgA2 resulted in a significantly smaller proportion of penetrating virions compared to PBS controls (Fig 4A). All of these results can be found in Figure 4. Known, albeit small, differences in binding of dIgA1 and dIgA2 could change the penetration even though they have the same specificity. We conclude that HGN194 dIgA1 and HGN194 dIgA2 can alter the distribution of HIV particles after rectal instillation based on the statistical analysis presented in Figure 4 and Figure 5B/C.

.

4. Is there any way to demarcate the edges of the epithelium in the rectal images so that we can more easily see the depth by eye?

Thank you for this useful suggestion to help the reader interpret the micrographs. We have added dashed, white lines to Figure 3 to demarcate the edges of the epithelium.

5. Table does not make clear which of the macaques are shown in figure 2 (with the exception of A15X085, which is stated in Table 1 as being evaluated by CT but discussed in text as being evaluated by PET??)

Thank you for this comment. All animals received PET imaging, which is either supported by MRI or CT. Animals received either MRI or CT depending on which location the experiment took place. All PET/MRI experiments took place at RII-UTSHA, while PET/CT experiments took place at NIRC. To make this clearer, we have included "PET/MRI" and "PET/CT" on Table 1 and added the following text.

"Therefore, positron emission tomography (PET) allows an unbiased visualization of the radiolabeled molecule at the whole animal level with the additional ability to have multiple scans to provide insights into kinetics and dynamics of distribution. Interpretation of the PET signal can be facilitated by coupled with an imaging modality that can reveal anatomical structures by imaging bones and tissues utilizing imaging approaches such as 3D x-rays through computerized tomography (CT) or the use of magnetic resonance imaging (MRI)." *(page 8, lines 148-154)*

Another major issue is whether there are any important differences associated with utilizing either PET or CT? These are discussed interchangeably in the text, but are the results actually interchangeable, or were there some interesting differences that could be discussed? Is one method superior to the other?

Thank you for this comment. For these studies, we were limited in access to either CT or MRI. There was access to the MRI at the RII-UTSHA but not CT, and there was access to CT at NIRC but not MRI. We have reviewed the manuscript and made our language more concise and indicated which animal received which supplemental imaging in Table 1. All animals received PET imagining, which was complemented and supported by either MRI or CT imaging. As we utilized them for interpreting the PET signal they are indistinguishable in this study.

"Interpretation of the PET signal can be facilitated by coupled with an imaging modality that can reveal anatomical structures by imaging bones and tissues utilizing imaging approaches such as 3D x-rays through computerized tomography (CT) or the use of magnetic resonance imaging (MRI)." (page 8, lines 151-154)

The finding of the virus at the mesenteric lymph nodes at 2 hours post viral administration is particularly interesting. Is there any way to evaluate which of the lymph node areas (T or B cells) is the location where the virions are being observed? Could you do immunofluorescence analysis with anti-CD3, CD4 or CD20 on adjacent section of the tissue? Was there any evidence for IgA being seen associated with those virions in the lymph node?

This is an excellent question. In Figure 5, the middle image of panel A shows virions (red) surrounded by dIgA1 (blue). We are also very interested in defining the localization of virions within the mesenteric lymph nodes. But such an analysis would require additional method development and is beyond scope of manuscript.

Part III – Minor Issues: Editorial and Data Presentation Modifications
Please use this section for editorial suggestions as well as relatively minor modifications of existing data that would enhance clarity.

Reviewer #1: Please explain in all figure legends what arrows are pointing to.

For all figures, we have explained what the arrows are pointing to in the figure legends.

The authors refer to rectal challenge studied in mice for the murine leukemia virus (line 401), but I couldn't find it in the cited paper. The paper describes s.c., i.v., and oral transmission, but not rectal challenge. It does describe virus arrival at mesenteric lymph nodes after oral uptake.

Thank you for this critical comment. We have corrected our mistake in the route of viral challenge in this specific reference.

Reviewer #2: Line 62 should read "Currently, an effective human immunodeficiency virus (HIV) vaccine does not exist".

This grammatical error has been corrected.

Line 104: "Notably, most vaccines do not stimulate protective IgA responses". Do we really know this, or do we assume this because IgA is seldom studied in vaccine trials?

Thank you for this question. Currently, robust IgA responses have been elicited from oral vaccines, such as the attenuated poliovirus and rotavirus vaccines. We have updated the following text to reflect this (line 104).

"To date, robust IgA responses have been found to be elicited by oral vaccines, such as the attenuated poliovirus and rotavirus vaccines(13, 14)."

Line 229 - a word seems to be missing ("to consider whether the entire colon needs to be excised")

This error has been corrected.

Line 262 - for clarity, text should read "but Cy5 signal was apparent in rectum, descending..."

We have made this correction for clarity.

Lines 372-375, wording could be clearer.

We have reworded this sentence for clarity.

Line 381: predominantly

This spelling error has been corrected.

Lines 407-9: Should read "while HGN194 dIgA2 protected 1/6 animals"

This error has been corrected.

Lines 413-414: "The issue...would be moot (not mute) if the virions within the immune complexes are inactivated or neutralized".

This word choice error has been corrected.

Reviewer #3: Some of the minor issues:
1. What is meant by this sentence at line 386: "It may be possible that dIgA2 has a greater capacity to form immune complexes with pathogens in the colon, specifically for shuttling to mesenteric lymph nodes and further inside the colon than dIgA1."? Are you saying that immune complexes with IgA1 are not forming due to the environment (pH manybe)? Could it be that immune complexes are forming but receptors for the antibody are not present? updated sentence

Thank you for this comment. We have clarified our language in the manuscript regarding this component in the discussion. We hope the following text that has replaced the sentence in question will be clearer for the reader.

"Our data suggests that immune complexes of the virus and HGN194 dIgA2 are more mobile relative to similar immune complexes formed with HGN194 dIgA1. This interpretation is supported by our observation that the viral particles appear more efficiently in the mesenteric lymph nodes and more abundantly in the transverse colon in the presence of HGN194 dIgA2 compared to HGN194 dIgA1. This increase mobility could be facilitated by HGN194 dIgA2 engagement of virus in the rectal environment or through interactions with receptors on resident cells." (page 20, lines 416-422)

2. Figure 4, text in manuscript is listed as A-C, Figure has A-I. change to a-c

The figure legend and manuscript has been corrected to A-I to match the figure.

3. Line 408, HGN194dIgA1 is repeated, I think one should be A2

This error has been corrected.

4. Line 423, Possibly include a discussion of the finding from the STEP HIV vaccine trial where they observed a correlation between serum IgA levels and increased risk of HIV transmission in those that were vaccinated.

We added a sentence to increase clarity for the reader relating to the finding from the RV144 trial regarding the correlation of serum IgA levels and HIV infection.

"The RV144 trial showed increased binding of Env-specific IgA in the plasma directly correlated to infection in the study participants,(37) fostering the idea that IgA enhanced viral infection." (page 21, lines 445-447)

5. Line 735, typo 'dD'

This error has been fixed.

Sincerely yours,

Thomas Hope, Ph.D.

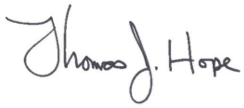

Professor, Northwestern University
Director, Viral Pathogenesis Core, Third Coast Center for AIDS Research
